# Supplementary material for: Increased Immunogenicity of Full-Length Protein Antigens through Sortase-Mediated Coupling on the PapMV Vaccine Platform
Source: Vaccines (Basel). 2019 Jun 12;7(2):49. doi: 10.3390/vaccines7020049 (PMC6630801; doi:10.3390/vaccines7020049)
Supplement: Supplementary file 1 [file vaccines-07-00049-s001.pdf]

Supplementary data

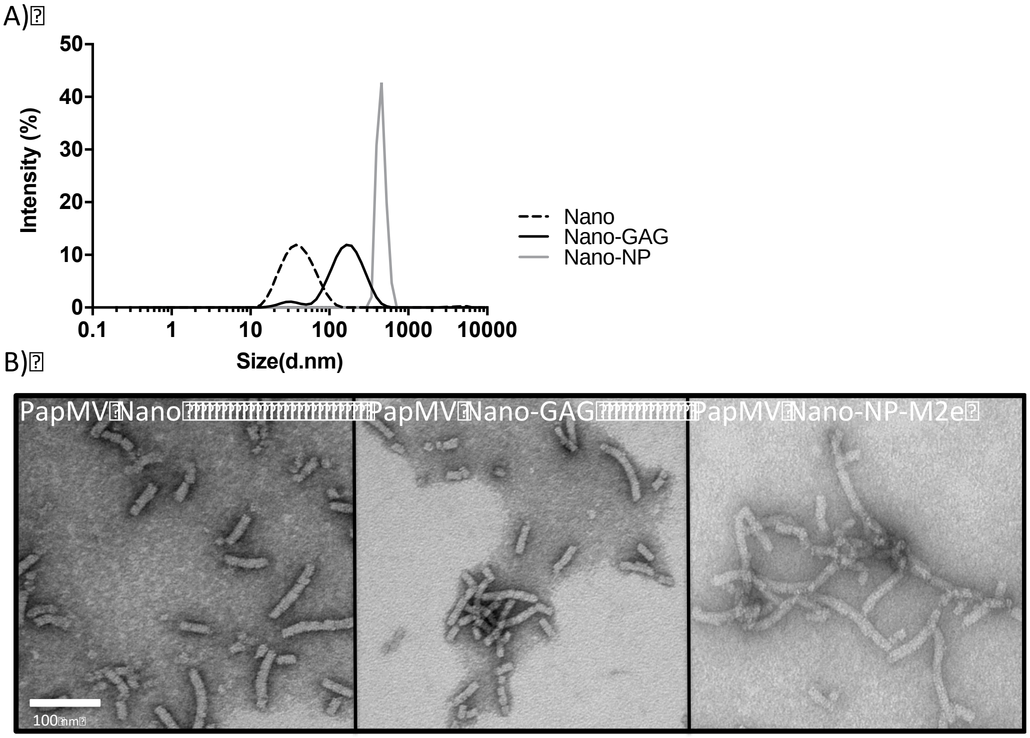

Figure S1. Characterization of the size and appearance of the PapMV nano coupled to the antigens. A) Dynamic ligth scattering (DLS) showing the length of the free PapMV nano, PapMV nano coupled to the GAG antigen and PapMV nano coupled to the NP-M2e antigen. The legend to identify the 3 samples is chowed beside the graph. B) Electron micrograph of the nanoparticles. The free PapMV nano are showed in the left panel and show the expected flexuous rod shape morphology. PapMV nano coupled to the GAG antigen is showed in the middle panel and the PapMV nano coupled to the NP-M2e antigen is showed in the left panel. The 100nm bar is showed in the left panel.

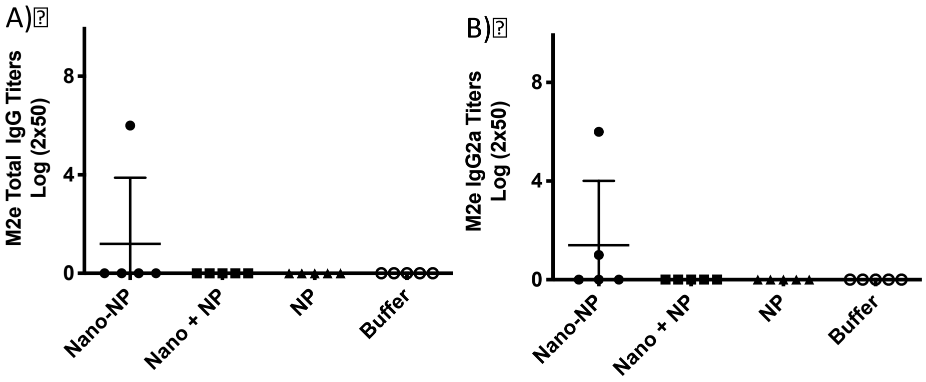

Figure S2. Assessment of the humoral response directed to the M2e antigen. Sera from the animals described at Fig. 3 was used to assess the humoral response directed to the M2e antigen of the immunized at day 28. The total IgG (A) and the IgG2a titers (B) were assessed by ELISA.
